# Systematic review on left atrial appendage closure with the LAmbre device in patients with non-valvular atrial fibrillation

**DOI:** 10.1186/s12872-020-01349-9

**Published:** 2020-02-12

**Authors:** Muhammad Ali, Angelos G. Rigopoulos, Mammad Mammadov, Abdelrahman Torky, Andrea Auer, Marios Matiakis, Elena Abate, Constantinos Bakogiannis, Stergios Tzikas, Boris Bigalke, Daniel Sedding, Michel Noutsias

**Affiliations:** 1 Mid-German Heart Center, Department of Internal Medicine III (KIM-III), Division of Cardiology, Angiology and Intensive Medical Care, University Hospital Halle, Martin-Luther-University Halle, Ernst-Grube-Strasse 40, D-06120 Halle (Saale), Germany; 2 3rd Department of Cardiology, Ippokrateio Hospital, Aristotle University of Thessaloniki, Konstantinoupoleos 49, 54642 Thessaloniki, Greece; 3 Department of Cardiology, Charité, Universitätsmedizin Berlin, Campus Benjamin Franklin, Berlin, Germany

**Keywords:** Anticoagulation, Atrial fibrillation, Closure device, LAmbre, Left atrial appendage, Left atrial appendage closure, Mortality, Prognosis, Stroke

## Abstract

**Background:**

Percutaneous closure (LAAC) of the left atrial appendage (LAA) is an efficacious preventive procedure for patients with non-valvular atrial fibrillation (NVAF) and considerable bleeding risk. We sought to systematically review the available LAAC data on the novel occluder device LAmbre™.

**Methods:**

For this systematic review, a search of the literature was conducted by 3 independent reviewers, reporting the safety and therapeutic success of LAAC in patients being treated with a LAmbre™. Publications reporting the safety and therapeutic success of LAAC using LAmbre™ in *n* > 5 patients were included.

**Results:**

The literature search retrieved *n* = 10 publications, encompassing *n* = 403 NVAF patients treated with a LAmbre™ LAAC, with relevant data regarding safety and therapeutic success of the procedure. The mean CHA_2_DS_2_-VASc Score was 4.0 + 0.9, and the mean HAS-BLED score was 3.4 + 0.5. The implantation success was 99.7%, with a mean procedure time of 45.4 ± 18.7 min, and a fluoroscopy time of 9.6 ± 5.9 min, and a contrast agent volume of 96.7 ± 0.7 ml. The anticoagulation regimen was switched to DAPT post procedure in the majority of the patients (96.8%). Partial and full recapture were done in 45.5% and in 25.6%, respectively. Major complications were reported in 2.9%, with 0.3% mortality, 1.7% pericardial tamponade, 0.3% stroke, and 0.6% major bleeding complications; no device embolization was observed. During follow up at 6 or 12 months, major adverse cardiovascular events were reported in 3.3%: Stroke or TIA in 1.7%, thrombus formation on the device in 0.7%, and residual flow > 5 mm in 1.0%. In some publications, the favorable implantion properties of the LAmbre™ for difficult anatomies such as shallow or multilobular LAA anatomies were described.

**Conclusions:**

This systematic review on the LAmbre™ LAA-occluder including *n* = 403 NVAF patients demonstrates an excellent implantion success rate, promising follow-up clinical data, and favorable properties for also challenging LAA anatomies,. While its design seems to be helpful in preventing device embolization, pericardial tamponade may not be substantially reduced by the LAmbre™ as compared with other established LAAC devices. Further larger prospective multicenter registries and randomized trials are needed to scrutinize the value of the LAmbre™ compared with established LAAC devices.

## Background

Atrial fibrillation (AF) is the most common prevalent cardiac arrhythmia. The non-valvular AF (NVAF) is associated with a significantly increased risk of embolic stroke and implicated as the second most common etiology for cerebral stroke with substantial morbidity and mortality especially in the aged population. AF patients with relevant risk for thromboembolic stroke should be treated with long-term oral anticoagulation (OAC) [1]. For NVAF patients with a substantially elevated risk for stroke and contraindications for OAC, it is recommended to consider the interventional closure of the left atrial appendage (LAAC) [1–3]. LAAC using percutaneous, catheter-based methods was first performed in 2001 with the PLAATO™ device [4]. At present, percutaneous LAAC is carried out most often using the Watchman™ (Boston Scientific, Marlborough, MA, USA) or the Amplatzer Amulet™ (St. Jude Medical, Saint Paul, MN, USA) devices [3]. While the overall implantion success rates increased, major adverse event rates decreased in recent years in large real-world prospective registries with these leading LAAC devices to 2.7–3.3% for the Watchman™ [5, 6], and 3.2% for the Amulet™, respectively [7].

Novel devices might enhance the procedure in some anatomically and technically complicated cases, and may thus improve the clinical results. The LAmbre™ (Lifetech Scientific Corp., Shenzhen, China) is a fully recapturable and repositionable LAA occluder, which has a relatively slim delivery system (8–10 French delivery sheath) compared with those of the Amulet™ (12–14 F) and the Watchman™ (14 F). It is basically constructed from nitinol mesh and polyester membranes. “LAmbre” is deduced from “an umbrella in the left atrial appendage” [8, 9]. It has obtained the CE Mark on June 16th, 2016. The main characteristics of the LAmbre™ device comprise the special stabilization system with U-shaped anchors / hooks, which target the trabeculae and pectinate muscles of the LAA [10]. Its main advantages include the construction-related reduced risk of LAA perforation due to the characteristic umbrella (U-shaped anchors) and the hooks, which act synergistically to prevent from LAA perforation. The sequences of device implantation include a straightforward roadmap with positioning the delivery system in the proximal area of the LAA, and next with deployment of the umbrella (8 U-shaped anchors covered with a membrane) in the LAA. After that, the whole LAmbre™ system is pushed gently forward towards the LAA for commitment of the stabilizing hooks into the trabeculae of the LAA wall, and finally, the cover is deployed to seal LAA orifice, again with a gentle push forward if needed [10]. The following tug test confirms device stability, and sealing of the LAA orifice can be confirmed by contrast agent injection via the delivery system, and by transesophageal echocardiography (TEE). The marked adaptability of the LAmbre™ device is based in major parts on the variable combinations of different sizes of the cover and of the umbrella, which renders the LAmbre™ device feasible for virtually all LAA anatomies with LAA orifice diameters > 12 mm. The articulated waist acts as compliant connecting point between the 2 components, allowing both parts to self-orient to the underlying anatomy, even in challenging anatomies, in multilobe LAA and in LAA with large orifice and relatively shallow LAA body [10, 11]. It has also been hypothesized that the shape of the umbrella of the LAmbre™ may be applicable to patients with LAA thrombus, since the umbrella will not seat deeply, and will therefore not mobilize, but rather isolate the LAA thrombus in the apex of the LAA.

This systematic review was undertaken to summarize the clinical data by the available publications on the LAmbre™ device, and to address potential new issues for future registries and clinical trials.

## Methods

**Fig. 1:**
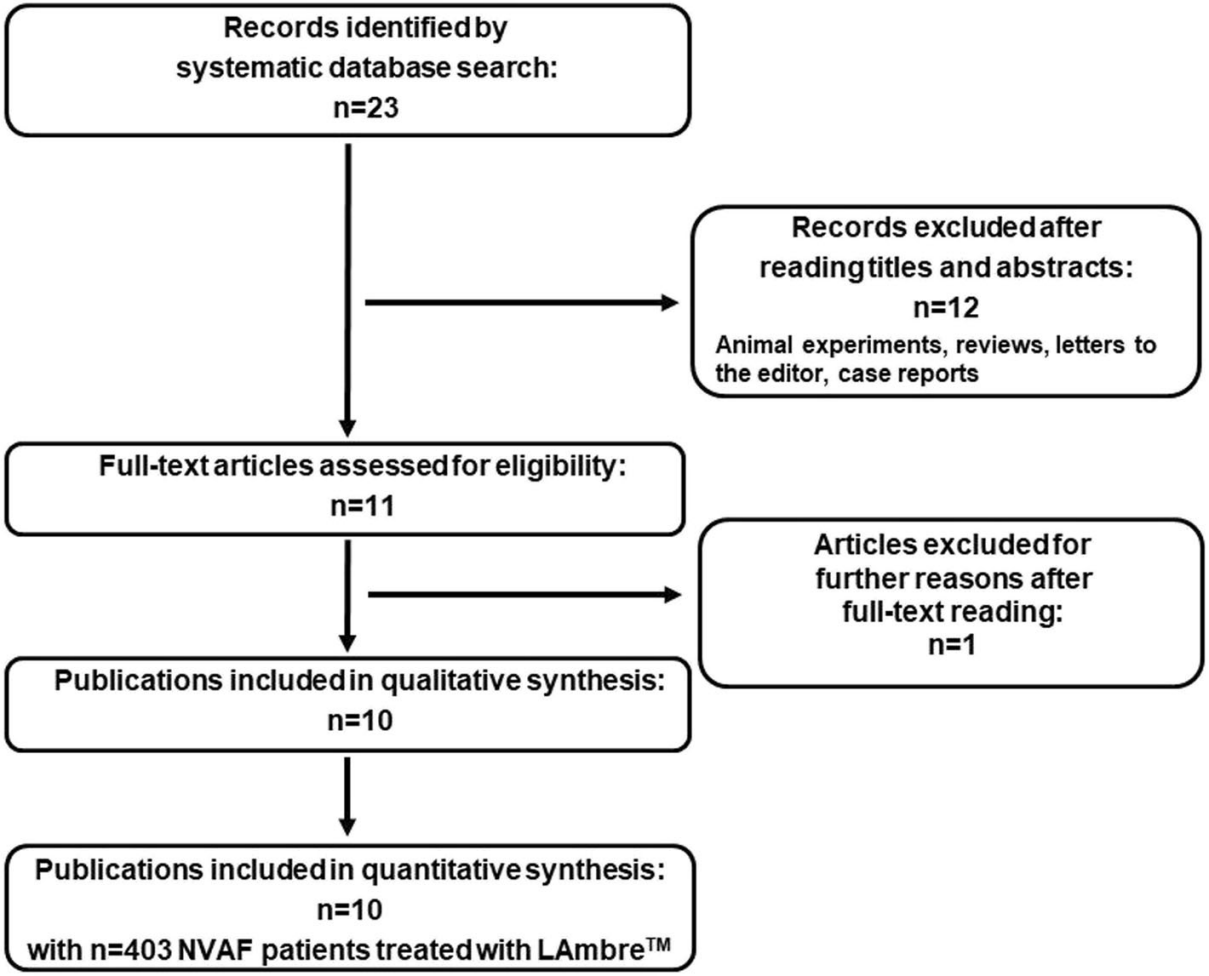
Study selection process for the systematic review

Electronic searches were carried out using Medline (via PubMed), Web of Science, the Cochrane Library and Embase following the PRISMA (Preferred Reporting Items for Systematic Reviews and Meta-Analyses) statement [12]. The databases were searched by 3 independent reviewers (MA, MM and MN; between inception and May 1st, 2019). We conducted a systematic search of the literature using the following combination of keywords / MeSH terms: “LAmbre device OR LAmbre occluder OR LAmbre atrial occluder”. Congress abstracts were discarded. The literature search was conducted using EndNote Version X8.2 (Thomson Reuters). Publications were judged relevant to the specific subject reporting the safety and therapeutic success of LAAC in *n* > 5 patients being treated with a LAmbre™. We excluded publications referring to only animal experiments or in vitro experiments, human studies on < 5 patients, case reports, congress reports, review articles, editorial letters, and publications written in languages other than English or German. However, data derived from case reports or case series reporting rare or focusing on particular complications were also considered for reporting the results and for the discussion, but were not included in the systematic data analysis. There were no discrepancies between the 3 reviewers. The study selection process is illustrated in the flow chart in Fig. 1. Finally, *n* = 10 publications on results of trials and registries were deemed eligible being included in this systematic review. We computed the investigational data in a Microsoft Excel datatable for statistical analysis (evaluation of means and standard deviations / SD).

## Results

The defined literature search revealed *n* = 23 entries of peer reviewed publications. Eleven publications were excluded according to the criteria described in the Methods section. In detail, n = 10 publications were excluded because they were case reports, and one further publication was a review. After full-text reading, an additional publication was excluded since it was a benchmark testing, without reporting clinical data [13].

The 10 publications included in the data analysis of this systematic review were published from 2016 until 2019 [9, 14–22]. The reported total cohort in these 10 publications comprised *n* = 403 NVAF patients, treated with implantation of a LAmbre™ device. The inclusion and exclusion criteria, the demographic data, the respective CHA_2_DS_2_-VASc and HAS-BLED scores, the procedural and complication data, as well as the OAC and antiplatelet treatment regimens are summarized in Tables 1-4. The patients had a mean age of 73.6 + 4.0 years, and 58.3% were males. 6 studies with 163 patients had a single-center design, while 4 studies included 240 patients from more than 1 center (number of participating centers ranging from 2 to 12). All publications were derived from open-label, non-randomized registries. 7 studies had prospective design, while 1 publication had retrospective study design. The remaining publications did not specify this issue. The studies were carried out in European countries or in China. The main goal of 8 investigations was to ascertain safety, feasibility, and efficacy of the LAmbre™ LAAC, while 2 publications studied primarily ECG and echocardiographic parameters [14], and the value of 3D-TEE guidance [16] in LAmbre™ LAAC procedures, respectively. The inclusion criteria comprised usually intolerance to long-term OAC, while being indicated in these NVAF patients with a high risk for thromboembolic complications (mean CHA_2_DS_2_-VASc Score: 4.0 + 0.9) and a high risk for major bleeding events under OAC (mean HAS-BLED: 3.4 + 0.5; Table 2). Referring to the exclusion criteria, however, a larger variety was listed in addition to known general contraindications for LAAC (i.e. clinical conditions not allowing TEE, LAA thrombus), and certain thresholds of LAA orifice diameter < 12 mm in 4 publications. In 5 publications, patients with LVEF< 30% or LVEF< 30% were excluded. Additionally, patients with LVEF< 40% were excluded in one further study (Table 1).

**Table 1:** Included patients, inclusion / exclusion criteria and demographic data

	Study type	Main goal of the study	Inclusion criteria	Major exclusion criteria	No. of patients subjected to implantation of LAmbre®; (No. of centers)	Age (years); (mean + SD)	Males (*n*= / %)
Chen et al. (2019) [21]	Open-label, non-randomized, prospective, single-center study	Safety, feasibility, and efficacy of LAAC with the LAmbre device in NVAF patients	NVAF, age > 18 years, CHA_2_DS_2_-VASc Score > 2, contraindication or intolerance to long-term OAC, refusal of OAC treatment, isolated noncontractile LAA after multiple AF ablation procedures	clinical conditions not allowing TEE and sedation; poor peripheral vessel access not allowing device delivery; LAA thrombus (TEE); LVEF< 30%; patients with atrial septal occluder; stroke or TIA in the past 30 d; acute myocardial infarction or unstable angina; decompensated heart failure (New York Heart Association functional class III–IV), or heart transplantation; rheumatic, significant degenerative, or congenital valvular heart diseases, artificial heart valve replacement operation; significant and unexplained pericardial effusion (≥4 cm^2^)	*n* = 30 (1 center; Germany)	77.6 ± 8.9	*n* = 15 / 50%
Feng et al. (2019) [22]	Open-label, non-randomized, prospective, single-center study	Safety, feasibility, and efficacy of LAAC with the LAmbre device in NVAF patients with or without prior ablation	NVAF, CHADS_2_ Score > 1, contraindication for or failure of OAC	LAA orifice diameter < 12 mm; LAA thrombus (TEE); LVEF< 30%; stroke or TIA in the past 30 d: presence of thrombus in the heart; prosthetic valve; myocardial infarction or unstable angina; acute infective endocarditis; pregnancy; symptomatic carotid artery disease; malignancies with an estimated life expectancy of ≤2 years; allergy to nitinol	*n* = 17 (1 center; China)	71.4 ± 7.8	*n* = 7 / 41.2%
Reinsch et al. (2018) [19]	Retrospective analysis of single-center case series	Safety, feasibility, and efficacy of LAAC with the LAmbre device	NVAF, CHA_2_DS_2_-VASc Score > 2, HAS-BLED > 3, past bleeding	N/A	n = 11 (1 center; Germany)	72.6 ± 7.9	*n* = 7 / 63.6%
Park et al. (2018) [18]	Open-label, non-randomized, prospective, multicenter study	Safety, feasibility, and efficacy of LAAC with the LAmbre device	NVAF> 3 months, > 18 years age, CHA_2_DS_2_-VASc Score > 2, and contraindications for OAC	LAA orifice diameter < 12 mm or > 30 mm, LAA diameter > 65 mm, LAA thrombus (TEE); LVEF< 30%, NYHA III or IV, prior heart transplantation; heart rate > 110 beats per minute; stroke or TIA in the past 30 d; past LAAC or surgical LAA removal, past ASD closure; rheumatic, degenerative or congenital valvular heart disease; recent or acute myocardial infarction or unstable angina; mechanical valve prosthesis; scheduled electrophysiological ablation procedure, scheduled pharmacological or electrical cardioversion; pre-procedural pericardial effusion; signs or symptoms of infection; pregnancy or breastfeeding; symptomatic carotid artery disease; malignancies with an estimated life expectancy of ≤2 years; allergy to nitinol, thrombocytopenia (platelet ≤100.000 per microliter); complex atherosclerotic plaques (≥4 mm) in the ascending aorta	*n* = 60 (2 centers; Germany)	74.4 ± 9.0	*n* = 40 / 66.7%
Kleinecke et al. (2018) [17]	Open-label, non-randomized, multicenter observational study	Safety, feasibility, and efficacy of LAAC with the LAmbre device and the FuStar steerable sheath	Common indications for LAAC, patients` explicit choice	LAA thrombus (TEE); planed cardiac surgery; history of ASD closure; endocarditis, active infections	n = 20 (2 centers; Germany)	76.6 ± 8.4	*n* = 12 / 60%
Cruz-Gonzalez et al. (2018) [20]	Open-label, non-randomized, prospective, multicenter observational registry	Safety and feasibility of LAAC with the LAmbre device	Common indications for LAAC	N/A	n = 7 (3 centers; Spain)	78.6 + 6.9	n = 3 / 42.9%
Chen et al. (2018) [9]	Open-label, non-randomized, prospective, single-center observational study	Safety, feasibility and efficacy of LAAC with the LAmbre device	Common indications for LAAC	N/A	n = 30 (1 center; Germany)	77.6 ± 8.9	*n* = 15 / 50.0%
Zhou et al. (2017) [16]	Open-label, non-randomized, prospective, single-center observational study	Value of 3D-TEE guidance of LAAC (LAmbre and Lefort)	NVAF, CHA_2_DS_2_-VASc Score > 2, contraindications for OAC	LAA thrombus (TEE); LVEF< 40%, NYHA IV; myocardial infarction within the last 3 months; vascular abnormalities interfering with LAAC	n = 21 (1 center; China)	66.6 ± 8.5	n = 15 / 71.4%
Huang et al. (2017) [15]	Open-label, non-randomized, prospective, multicenter study (NCT02029014)	Safety, feasibility, and efficacy of LAAC with the LAmbre device	NVAF > 18 years age with CHADS_2_ Score > 1 and not suitable for long term OAC/VKA	LAA orifice diameter < 12 mm; LAA thrombus (TEE); LVEF< 30%, NYHA IV; stroke or TIA in the past 30 d; past ASD closure; infective endocarditis; hemorrhagic disease; pregnancy; endocarditis; prosthetic valve	*n* = 153 (12 centers; China)	69.3 + 9.4	*n* = 87 / 56.2%
Jie et al. (2016) [14]	Open-label, non-randomized, single-center observational study	Investigation on changes of ECG and echocardiographic parameters after LAAC	NVAF, history of previous bleeding, high risk of bleeding, inability to adhere with OAC	LAA orifice diameter < 12 mm or > 30 mm; LAA thrombus (TEE); LVEF< 30%; mitral valve stenosis	*n* = 54 (1 center; Germany)	71.1 ± 9.1	n = 34 / 63.0%
Total / Overall mean					403	73.6 + 4.0	*n* = 235 / 58.3%

§: 1 enrolled patient did not undergo LAAC due to the anatomy of the interatrial septum not suitable for transseptal puncture

Abbreviations

*ABL* Acute brain lesions

*ASD* Atrial septum defect

*IAS* Interatrial septum

*LAA* Left atrial appendage

*LAAC* Closure of the left atrial appendage

*MRI* Magnetic resonance imaging

*N/A* Not available

*NOAC* New oral anticoagulants

*NVAF* Non-valvular atrial fibrillation

*OAC* Oral anticoagulation

*VKA* Vitamin K antagonists

**Table 2:** Implant success rates, scores (CHA_2_DS_2_-VASc or CHADS_2_ and HAS-BLED), procedural data and anesthesia type

	CHA_2_DS_2_-VASc or CHADS_2_ Score	HAS-BLED Score	Implantion Success (%)	Mean procedure time (min)	Mean fluoroscopy time (min)	Contrast agent (ml)	Radiation dose (cG*cm^2^)	Anesthesia type (n=/%)	LVEF; (mean + SD)
Chen et al. (2019) [21]	CHA_2_DS_2_-VASc: 3.9 ± 1.5	4.1 + 1.0	100%	29.0 ± 10.1	3.5 ± 1.9	N/A	N/A	CS: *n* = 30/100%	N/A
Feng et al. (2019) [22]	CHADS_2_: 2.5 ± 1.1	2.7 + 0.8	100%	67.2 ± 11.9	18.6 ± 14.4	N/A	N/A	N/A	63.6 ± 4.4
Reinsch et al. (2018) [19]	CHA_2_DS_2_-VASc: 3.3 + 1.0	3.0 + 1.0	100%	65.1 ± 27.2	7.1 ± 2.7	< 50	N/A	CS: *n* = 11/100%	48.5+ 9.1
Park et al. (2018) [18]	CHA_2_DS_2_-VASc: 4.0 + 1.6	3.2 + 1.3	100% ^a^	33.9 ± 17.6	12.7 ± 4.8	97.2 ± 44	N/A	CS: *n* = 23/38.3% General: *n* = 37/61.7%	58.0 ± 6.9
Kleinecke et al. (2018) [17]	CHA_2_DS_2_-VASc: 5.0 + 2.0	3.7 + 1.3	100%	23.4 ± 9.2	11.9 ± 4.1	96.2 ± 45.7	2718.4 ± 3835.3	N/A	56.4 ± 9.2
Cruz-Gonzalez et al. (2017) [20]	CHA_2_DS_2_-VASc: 5.4 + 1.0	3.3 + 0.8	100%	N/A	N/A	N/A	N/A	N/A	N/A
Chen et al. (2018) [9]	CHA_2_DS_2_-VASc: 3.9 + 1.5	4.1 + 1.0	100%	29.0 ± 10.1	3.5 ± 1.9	N/A	N/A	N/A	N/A
Zhou et al. (2017) [16]	CHA_2_DS_2_-VASc: 3.9 + 1.3	N/A	100%	49.7 ± 9.1	N/A	N/A	N/A	N/A	53.9 ± 6.1
Huang et al. (2017) [15]	CHA_2_DS_2_-VASc: 4.0 + 1.7	N/A	99.3% (*n* = 152/153)	66.0 ± 24.0	N/A	N/A	N/A	General: n = 152/100%	N/A
Jie et al. (2016) [14]	CHADS_2_: 2.4 ± 1.3	N/A	N/A	N/A	N/A	N/A	N/A	N/A	61.0 ± 8.5
Total / Overall mean	CHA_2_DS_2_-VASc: 4.0 + 0.9	3.4 + 0.5	99.7% (*n* = 348/349)	45.4 ± 18.7	9.6 ± 5.9	96.7 ± 0.7		CS: *n* = 64/25.2% General: *n* = 189/74.4%	56.9 ± 5.3

^a^: 1 enrolled patient did not undergo LAAC due to the anatomy of the interatrial septum not suitable for transseptal puncture

Abbreviations

*CS* Conscious sedation

*LVEF* Left ventricular ejection fraction

*N/A* Not available

**Table 3:** Pre- and postprocedural regimens, partial or full recapture of the device, resizing of the device

	Device for transseptal puncture (*n*= / %)	Preprocedural antiplatelet regimens or OAC (VKA, NOAC); (*n*= / %)	Regular antiplatelet or OAC regimens post-LAAC procedure	Partial recapture of the device (*n*= / %)	Full recapture of the device (*n*= / %)	Resizing of the device (*n*= / %)
Chen et al. (2019) [21]	N/A	VKA: *n* = 13 / 56.7%; NOAC: *n* = 17 / 1.7%	OAC: *n* = 1 / 3.3% DAPT for 6 months, followed by lifetime ASS: *n* = 29 / 96.7%	14 / 46.7%	N/A	0
Feng et al. (2019) [22]	Brockenbrough needle and SL1 transseptal sheath (St. Jude Medical)	N/A	DAPT for 3 months, followed by lifetime ASS	N/A	N/A	1 / 5.9%
Reinsch et al. (2018) [19]	Brockenbrough needle and SL1 transseptal sheath (St. Jude Medical)	N/A	DAPT for 6 months, followed by lifetime ASS	11 / 27%	1 / 9%	1 / 9%
Park et al. (2018) [18]	N/A	ASS: *n* = 46 / 78%; Clopidogrel: *n* = 49 / 82%; heparin: *n* = 14 / 23%; VKA: *n* = 2 / 3.3%; NOAC: *n* = 1 / 1.7%	DAPT for 3 months	21 / 38%	9 / 14%	3 / 5%
Kleinecke et al. (2018) [17]	Brockenbrough needle (St. Jude Medical)	N/A	N/A	N/A	20 / 100%	0
Cruz-Gonzalez et al. (2018) [20]	N/A	N/A	DAPT: *n* = 3 / 43% SAPT: *n* = 1 / 14% NOAC: *n* = 2 / 28% LMWH: *n* = 1 / 14%	N/A	N/A	N/A
Chen et al. (2018) [9]	N/A	N/A	DAPT: *n* = 29 / 96.7%	N/A	1 / 3.3%	N/A
Zhou et al. (2017) [16]	N/A	VKA: *n* = 21 / 100%	N/A	N/A	N/A	N/A
Huang et al. (2017) [15]	N/A	N/A	N/A	N/A	N/A	N/A
Jie et al. (2016) [14]	Brockenbrough needle with SL1 transseptal sheath (St. Jude Medical)	ASS: *n* = 13 / 18% Clopidogrel: *n* = 5 / 7% VKA: *n* = 18 / 25%	N/A	N/A	N/A	N/A
Total / Overall mean	Brockenbrough needle, mostly used with the SL1 transseptal sheath	VKA or NOAC: *n* = 72/165 = 43.6% Other: *n* = 93/165 = 56.4%	DAPT in *n* = 150/155 = 96.8%	46 / 45.5%	31 / 25.6%	5 / 3.6%

Abbreviations

*DAPT* Dual antiplatelet therapy (aspirin plus clopidogrel)

*LMWH* Low-molecular weight heparin

*N/A* Not available

*NOAC* New oral anticoagulants

*OAC* Oral anticoagulation

*SAPT* Single antiplatelet therapy (aspirin or clopidogrel)

*VKA* Vitamin K antagonists

**Table 4:** Pre- and postprocedural regimens, complications and outcomes

	Major procedure-related complications (*n*= / %)	Minor procedure-related complications (*n*= / %)	Follow up time / Outcome (AE) (*n*= / %)
Chen et al. (2019) [21]	Total: *n* = 0 / 0%	0%	6 months: Total: *n* = 0 / 0%; Stroke/TIA: *n* = 0 / 0%; TFD: *n* = 0 / 0%; RF > 5 mm: *n* = 0 / 0%; Major Bleeding: *n* = 0 / 0%; Device embolization: *n* = 0 / 0% RF < 5 mm: *n* = 9 / 30.0%; Minor Bleeding: *n* = 0 / 0%
Feng et al. (2019) [22]	Total: *n* = 1 / 5.9%; Death: *n* = 0 / 0%; PerTam: *n* = 1 / 5.9%	*n* = 2 / 11.8% (haematoma)	12 months: Total: *n* = 0 / 0%; Stroke/TIA: *n* = 0 / 0%; TFD: *n* = 0 / 0%; RF > 5 mm: *n* = 0 / 0%; Major Bleeding: *n* = 0 / 0%; Device embolization: *n* = 0 / 0% RF < 5 mm: *n* = 2 / 11.8%; Minor Bleeding: *n* = 0 / 0%; n = 1 / 5.9% sudden cardiac death at 545 days
Reinsch et al. (2018) [19]	Total: *n* = 0 / 0%; Death: *n* = 0 / 0%	0%	6 months: Total: *n* = 0 / 0%; Stroke/TIA: 0%; TFD: 0%; RF > 5 mm: 0%; Major Bleeding: *n* = 0 / 0%; Device embolization: *n* = 0 / 0% RF < 5 mm: 0%; Minor Bleeding: *n* = 0 / 0%
Park et al. (2018) [18]	Total: *n* = 4 / 6.7%; Death: *n* = 1 / 1.7%; PerTam: *n* = 3 / 5.0%; PerTam requiring surgery with fatal outcome: *n* = 1 / 1.7%; Pseudoaneurysm of right AFC requiring surgical repair: *n* = 1 / 1.7%	0%	12 months: Total: *n* = 5 / 6.7%; Stroke/TIA: *n* = 2 / 1.6%; TFD: 0%; RF > 5 mm: *n* = 3 / 5%^a^; Major bleeding: 0%; Device embolization: *n* = 0 / 0% RF < 5 mm: *n* = 14 / 24.6%; Minor bleeding: *n* = 5 / 5% Death unrelated to LAAC: 3%
Kleinecke et al. (2018) [17]	Total: *n* = 0 / 0%; Death: *n* = 0 / 0%	0%	N/A
Cruz-Gonzalez et al. (2017) [20]	Total: *n* = 0 / 0%; Death: *n* = 0 / 0%	0%	N/A
Chen et al. (2018) [9]	Total: *n* = 0 / 0%; Death: *n* = 0 / 0%	0%	6 months: Total: n = 0 / 0%; Stroke/TIA: 0%; TFD: 0%; RF > 5 mm: 0%; Major Bleeding: *n* = 0 / 0%; Device embolization: *n* = 0 / 0%
Zhou et al. (2017) [16]	Total: *n* = 0 / 0%; Death: *n* = 0 / 0%	0%	N/A
Huang et al. (2017) [15]	Total: *n* = 5 / 3.3%; Death: *n* = 0 / 0%; PerTam: *n* = 2 / 1.3; Stroke: *n* = 1 / 0.7%; Major bleeding: *n* = 1 / 0.7%	*n* = 4 / 2.6% (Femoral hematoma: *n* = 2; Arteriovenous fistula: *n* = 1 Pseudoaneurysm; *n* = 1)	12 months: Total: *n* = 5 / 3.9%; Stroke: *n* = 3 / 2.0%; TFD: n = 2 / 1.3%; RF > 5 mm: 0%; Major Bleeding: *n* = 0 / 0%; Device embolization: *n* = 0 / 0% Death unrelated to LAAC: *n* = 1 / 1.3% Available TEE at 12 months: *n* = 121 No RF: *n* = 102 / 84.3%; RF 1–3 mm: *n* = 18 / 14.9%; RF > 3 mm: *n* = 1 / 0.8%
Jie et al. (2016) [14]	N/A	N/A	N/A
Total / Overall mean	Total: *n* = 10 / 2.9%; Death: *n* = 1 / 0.3%; PerTam: *n* = 6 / 1.7%; Stroke: *n* = 1 / 0.3%; Major bleeding / Vascular complications: *n* = 2 / 0.6%	Total: *n* = 6 / 1.7%	Total: *n* = 10 / 3.3%; Stroke/TIA: *n* = 5 / 1.7%; TFD: *n* = 2 / 0.7%; RF > 5 mm: *n* = 3 / 1.0%; Major Bleeding: *n* = 0 / 0%; Device embolization: *n* = 0 / 0%

^a^: After the detection of RF > 5 mm at TEE at 1 month (3 cases), DAPT was switched to OAC. In 2 patients with persisting RF > 5 mm after 12 months, successful percutaneous closure of the leak with an Amplatzer vascular plug was undertaken

Abbreviations

*AE*: adverse events

*AFC*: Arteria femoralis communis

*DAPT*: dual antiplatelet therapy (aspirin plus clopidogrel)

*N/A* Not available

*PC* Pericardiocentesis

*PE* Pericardial effusion

*PerTam* Pericardial tamponade

*RF* Residual flow

*TFD* Thrombus formation on the device

The reported mean LVEF was 56.9 ± 5.3%. The implantion success was 99.7%, with a mean procedure time of 45.4 ± 18.7 min, and a mean fluoroscopy time of 9.6 ± 5.9 min, with mean contrast agent volume of 96.7 ± 0.7 ml per LAAC procedure. The reported anesthesia type was conscious sedation (CS) in 25.2%, while general anesthesia was used in 74.4% of the reported patients (Table 2).

The device for transseptal puncture was the SL1 transseptal sheath using the Brockenbrough needle (St. Jude Medical). The OAC regime before LAAC was switched to DAPT post procedure in the majority of the reported patients (96.8%). Partial recapture was reported in 45.5%, full recapture was done in 25.6%, and resizing of the device was undertaken in 3.6% of the reported patients, respectively (Table 3).

The mean frequency of major complications was 2.9%, with 0.3% mortality, 1.7% pericardial tamponade (PerTam), 0.3% stroke, and 0.6% major bleeding complications. During follow up at 6 or 12 months, major adverse cardiovascular events were reported in 3.3%: Stroke or TIA in 1.7%, thrombus formation on the device (TFD) in 0.7%, and residual flow > 5 mm in 1.0%. No major bleeding or device embolization events were reported (Table 4).

Interestingly, the success rate of LAmbre™ implantation was 100% in all *n* = 30 patients, 66.7% of whom, however, had difficult chicken-wing LAA morphology, including 1 patient who had twice failed procedures previously using Watchman™ and Amulet™ device implantation because of the especially challenging LAA morphology [9]. 18% of the patients included in a further publication had been previously rejected for other LAAC devices due to challenging anatomy of the respective LAA (too shallow for the Watchman™ LAAC) [19]. A further multicenter observational feasibility and safety study reported 100% success rate without any periprocedural complications (0%) in *n* = 20 patients treated with the LAmbre™ LAAC using the FuStar steerable sheath [17]. In some publications, which were partly excluded from the systematic analysis, the favorable implantion properties of the LAmbre™ for difficult anatomies such as shallow or multilobular LAA anatomies, or in cases with LAA thrombus resistant to OAC, were described [11, 23]. The remaining retrieved case reports referred to experiences of LAmbre™ LAAC procedures with particular LAA anatomies. One report focused on a 72-year-old-woman with chicken wing LAA morphology with a large ostium being successfully treated with a LAA LAmbre™ [11]. A further case reported from the Centro Cardiologico Monzino in Italy on a 68-year-old man who underwent pulmonary vein isolation with cryoballoon treatment combined with LAAC using a LAmbre™ device described an extracardiac asymptomatic early post-implantation dislodgment of the device and embolization to the abdominal aorta at the level of the renal arteries. The dislodged LAAC device was retrieved by a percutaneous approach [24]. A further particular case reported a successful deployment of a LAmbre™ LAAC in a patient with documented thrombus in the LAA [23]. In this exceptional case, the patient presented with cerebral stroke after having discontinued OAC with direct inhibitor of factor Xa in the setting of a dental procedure. The chicken wing shaped LAA revealed a thrombus, and after discussion it was decided to conduct LAA occlusion using a LAmbre™ device, which was successful and without further cerebral embolism [23].

A prospective study evaluated the incidence of magnetic resonance imaging (MRI)-detected acute brain lesions (ABLs) as well as potential changes in neurocognitive function in *n* = 23 in AF patients after percutaneous LAAC using the Amulet™ (*n* = 18), Occlutech™ (*n* = 3), or LAmbre™ (n = 2) device. *N* = 37 ABLs were detected by MRI in about half of all patients (i.e. in 12 of 23 patients) after LAAC. The number of periprocedural LAA angiographies was significantly higher in patients with ABL than in those without ABL, and was associated with a higher number of ABLs (*p* = 0.048) [25]. However, after performing LAAC, the Montreal Cognitive Assessment (MoCa) test and the National Institutes of Health Stroke Scale (NIHSS) scores revealed similar results compared to the pre-LAAC assessment [25].. In a further analysis on *n* = 25 patients, the authors reported MRI-detected ABLs in *n* = 12/25 (48%) of the LAAC-patients. Importantly, the follow-up (FUP) MRI which was performed in *n* = 7 ABL patients 3 months after the LAAC-implantation, no residual ABLs were detectable in 71% of these patients. There were no significant changes in neurocognitive function (MoCA-test and NIHSS-score) either after LAAC or at the 3-month FUP [17, 26]. These publications included only 2 patients treated with a LAmbre™ it was not designed to report success and complication rates, and was therefore not considered for the evaluation of the systematic review data.

## Discussion

Former studies have reported that > 90% of thromboembolic structures related to NVAF originate from the LAA [27]. A relevant proportion of NVAF patients is difficult to treat with OAC, particularly due to their substantially increased risk of major bleeding complications [2]. The thrombogenicity of the LAA in NVAF patients provides the rationale for OAC, and for the interventional LAAC. The Watchman™ device [28] has proved its non-inferiority compared to OAC in the prevention of thromboembolic events, plus the advantage of the lower rate of hemorrhagic events [1]. The Amplatzer Cardiac Plug has also been extensively tested with satisfactory results as a method for the prevention of LAA thrombosis and of cardioembolic stroke in chronic NVAF patients [8, 27]. However, both these most frequently used LAAC devices are accompanied by disadvantages such as the need for relatively large delivery sheaths (12–14 French), and only limited potential for recapturing and for repositioning [10, 29]. The percutaneous LAAC requires considerable technical and theoretical skills as well as adequate training under the supervision of expert colleagues [29]. Imaging support with experienced TEE is of paramount importance to guide the procedure [30]. Additionally, cardiac computed tomography angiography (CCTA) is recommended for LAAC [3]. Moreover, the interventionalists must be experienced in managing the typical LAAC associated complications. In particular, PerTam is a life-threatening complication needing immediate pericardiocentesis [31, 32].

The initial clinical experience registered in the prospective, observational, cohort study of Chen at al [9]. confirmed the feasibility and safety of the LAmbre™ occluder for catheter-based LAAC among patients with AF who had high risk of stroke and contraindications for oral anticoagulants and presented excellent periprocedural and short-term clinical outcome data for stroke prevention without indication for re-hospitalization. Zhou et al. discussed the clinical value of RT-3D TEE in transcatheter LAAC and the indications of 2 LAAC devices including LAmbre™ [16]. The LAmbre™ device was especially recommended for the patients with (i) multilobe LAA with a relatively high crest inside, (ii) 2 lobes of close sizes in a bi-lobe LAA, and (iii) LAA depth of less than the ostial dimension or less than 21 mm suggesting different indications for different LAA-occluder-device design. However, the observational nature of the study, and the relatively small number of patients, as well as the lack of long-term follow up data are acknowledged limitations of this study [16]. The excellent results obtained in [20] were attributed by the author to the growing experience in percutaneous LAAC as well as the LAmbre™ design. We must also consider that these procedures had been performed in experienced centers. Unfortunately, the use of CCTA for preinterventional imaging was not reported in detail in the included publications. Since CCTA is currently recommended for LAAC patients [3], one may hypothesize that preinterventional CCTA might have had a beneficial impact on the resulting success rates of the LAmbre™ LAAC. Thus, these promising data need to be interpreted with caution, and we must await data from large-scale studies and registries reflecting the real-world situation, especially with regard to success rate and complications.

The data available in the published reports about LAAC in NVAF patients with thrombus present in the LAA using special techniques are sparse [33]. The first case of LAAC with the Conformité Européenne (CE) mark-obtaining LAmbre™ device in the presence of thrombus was reported in 2017. The characteristic composition of this 2-part-device could make this device ideal for LAAC with a thrombus present if the LAA, since it might potentially prevent thrombus migration [23]. For this particular condition, however, we need more data to confirm this specific indication. Percutaneous LAAC can be challenging in selected cases, such as in the presence of chicken wing morphology, in multilobe LAA, or in LAA with a large difference of size between the ostium and the body of the LAA.

A relevant advantage of the LAmbre™ device may derive from the combination of distal hooks and the U-shaped ends and the central waist design, which may aid to achieve complete sealing, and ultimately also to prevent dislodgement and embolization of the device [11]. An additional issue, which needs more scrutiny for the standardized comparison of the available published data on the various LAAC devices, is the delivery sheath. In a recent publication, the steerable FuStar sheath was used, which facilitates the coaxial alignment of the sheath to the individual angle and morphology of the LAA [17]. This may be of importance in light of the wide heterogeneity of the human LAA anatomies.

The occurrence of device-non-related complications and findings is also important to evaluate, which might reveal particular device-associated prevalences. As such, acute brain lesions (ABLs) have been reported for several cardiac interventional procedures and for LAAC [34, 35]. In one study analyzing ABLs also in LAmbre™ treated patients, ABLs were reported in 48% of the LAAC-patients. However, since no residual ABLs were detectable in 71% of these LAAC-treated patients at the 3-months-FUP examination, and since no significant neurocognitive alterations were discernable by the MoCA-test and the NIHSS-score, the clinical significance of these MRI-detected lesions is questionable [17, 26].

Finally, the seldom event of embolization of the LAAC device remains a complication which needs high patients numbers to observe. In a systematic review of 17 studies published on different LAAC devices, Bajaj et al. reported an embolization rate of 3.9% [24, 36]. After this report, further case reports described embolization of Watchman™ devices to the left ventricular outflow tract with subsequent destruction of the aortic leaflets [24, 37]. After the inefficient attempt of percutaneous extraction, this severe complication necessitated valve replacement. Therefore, early recognition of any possible device-related complication by early post-implantation in-hospital check of the device position by TEE is pertinent. Hypothetically, LAAC device embolization could be asymptomatic if dislodged into the left atrium or further locations (i.e. into the aorta) without immediate hemodynamic relevance. Because of the lack of consensus regarding the management of migrated devices, the method of retrieval is decided on an individual basis, according to the embolized device destination, the patient’s hemodynamic status, and on the operator’s experience [24, 38].

The reported peri-procedural and long term complications after LAmbre™ LAAC evaluated in this systematic review were comparably low and within the range of the published Watchman™ [39] and the Amulet™ LAAC registries [7]. In particular, no device embolization was reported, and the rate of TFD was very low (0.7%). TFD may be associated with residual peri-device leak [40], which is less likely to occur with the LAmbre™ LAAC due to its disc closing the LAA ostium (as opposed to the e.g. non-disc based LAAC such as the Watchman™ LAAC [41]. Furthermore, NVAF patients with LVEF< 30% or LVEF< 40% were excluded by the predefined study criteria in 6/10 publications included in this systematic review focusing on the LAmbre™ LAAC. Considering that low LVEF may be a contributing factor for low flow, and consequently for sludge formation in the LAA, which may hypothetically contribute to TFD, future studies are warranted to evaluate the prevalence of TFD and of further periprocedural complications of the LAmbre™ LAAC in NVAF patients with low LVEF. Device embolization occurred in 0.2% of the patients in the multicenter Watchman™ EWOLUTION registry [5], and in 0.1% of the patients in the global Amulet observational registry [7]. Although no far-reaching conclusions can be drawn so far in light of the limited experience with this fairly novel occluder, the umbrella consisting of 8 hooks stabilizing the device in the trabeculae of the LAA may be a unique advantage of the LAmbre™ device in this regard. An additional feature contributing to the low frequency of device embolizations may be attributed to the comparably high radial force of the LAmbre™ LAAC device [13].

The design of the umbrella of the LAmbre™ was also intended to decrease the rates of pericardial tamponades being associated with other LAAC devices. Nonetheless, the rate of pericardial tamponades of 1.7% for the LAmbre™ is in our systematic review not substantially lower, rather slightly higher compared with the data of the Watchman™ EWOLUTION (0.5%) and of the global Amulet observational registry (1.2%) [7]. Notwithstanding these data, one should also consider that the overall complication rates decreased substantially over time with the growing interventional experience e.g. for the Watchman™ LAAC (starting from 8.7% in the PROTECT-AF, and showing a continuous decrement over time to 2.7% in the EWOLUTION registry) [5]. The rate of major bleedings or vascular complications in this systematic review was 0.6% for the LAmbre™ device, which did not differ compared with the Watchman™ EWOLUTION (0.8%) and of the global Amulet observational registry (0.7%) [7]. These data do not support the notion that the slimmer delivery device for the LAmbre™ (8–10 F versus 12–14 F) may translate to substantially lower peri-procedural vascular or bleeding events. The reported procedure time (45.4 ± 18.7 min), fluoroscopy time (9.6 ± 5.9 min), and the total contrast media volume (96.7 ± 0.7 mL) were within the range reported in the Watchman™ [39] and the Amulet™ LAAC registries [7].

However, the long-term data of our systematic review for NVAF patients treated with LAmbre™ implantation may be hampered by the fact that different antiplatelet regimens were used in the publications reporting regular protocols: DAPT for either 3 or for 6 months. However, the heterogeneity of various OAC or antiplatelet regimens is a known source of vast heterogeneity in LAAC registries: the post-interventional regimens in a recently published multicenter Amulet™ LAAC registry on 1088 patients comprised a multitude of various treatments: no therapy, single antiplatelet treatment (aspirin or clopidogrel), dual antiplatelet treatment for 3, 6, or 12 months, or OAC with or without antiplatelet therapy [42].

Apart from the following 2 studies including *n* = 213 (53%) patients from 14 centers [15, 18], the remaining 8 studies included in this systematic review are largely based on single-center registries with only few included patients. Thus far, the available data on the LAmbre™ are not solid for definitive conclusions. Given the lack of high quality clinical data for the LAmbre™, further evidence is pertinent to foster a solid evidence for this LAAC device with its unique properties.

### Limitations of the study

The data of this systematic review may be hampered by multiple factors, especially by all known sources of bias being associated with non-randomized registries, especially in the case of single-center registries. Among others, underreporting of major adverse events may be an issue as compared with real-world data. No randomized controlled trial (RCT) is available comparing the LAmbre™ with further LAAC devices, or with continuation of OAC, so far. RCT for LAAC candidates are difficult to establish, since the potential study NVAF patients usually have known substantial complications under OAC to justify the peri-procedural risks of LAAC. The use of various inclusion and exclusion criteria used for the different publications in our systematic review imposes substantial heterogeneity to the cumulated data. Since patients with LVEF< 40% were excluded in several studies, resulting in a mean LVEF of 56.9%, no relevant conclusions can be drawn for NVAF patients with reduced LVEF. Finally, a varying magnitude of parameters evaluated in our systematic review were not reported in some of the included publications.

## Conclusions

The LAmbre™ has entered the LAAC scene with a convincing cumulative evidence. Based on the available published data, the LAmbre™ LAAC has been proven as an effective and safe LAAC device for NVAF patients with a high risk for stroke and concomitant high bleeding risk or further contraindications for OAC. However, it is obvious that the use of such novel LAAC devices by highly experienced staff require careful selection of eligible patients and detailed characterization of the individual LAA morphology for both increasing the success rates and for reducing periprocedural complication rates. We need data from large-scale studies and registries reflecting the real-world situation, especially with regard to the success rates and complications, as well as to the specific advantages and disadvantageous characteristics of the respective LAAC devices.

## Data Availability

All data implemented in this systematic review are presented within the manuscript.

## Abbreviations

ABL: Acute brain lesion(s)
AE: Adverse events
AF: Atrial fibrillation
ASD: Atrial septum defect
CCTA: Cardiac computed tomography angiography
CS: Conscious sedation
DAPT: Dual antiplatelet therapy (aspirin plus clopidogrel)
FUP: Follow up
IAS: Interatrial septum
LAA: Left atrial appendage
LAAC: LAA closure
LMWH: Low-molecular weight heparin
LVEF: Left ventricular ejection fraction
MRI: Magnetic resonance imaging
NOAC: New oral anticoagulant(s)
NVAF: Non-valvular atrial fibrillation
OAC: Oral anticoagulation
PC: Pericardiocentesis
PE: Pericardial effusion
PerTam: Pericardial tamponade
RF: Residual flow
SAPT: Single antiplatelet therapy (aspirin or clopidogrel)
TEE: Transesophageal echocardiography
TFD: Thrombus formation on the device
VKA: Vitamin K antagonists
